# Reversible Deposition and Stripping of the Cathode Electrolyte Interphase on Li_2_RuO_3_

**DOI:** 10.3389/fchem.2020.00681

**Published:** 2020-08-04

**Authors:** Julia C. Hestenes, Andrew W. Ells, Mateo Navarro Goldaraz, Ivan V. Sergeyev, Boris Itin, Lauren E. Marbella

**Affiliations:** ^1^Department of Applied Physics and Applied Mathematics, Columbia University, New York, NY, United States; ^2^Department of Chemical Engineering, Columbia University, New York, NY, United States; ^3^Bruker Biospin Corporation, Billerica, MA, United States; ^4^New York Structural Biology Center, New York, NY, United States

**Keywords:** cathode-electrolyte interphase (CEI), Li_2_RuO_3_, Li-excess cathode, Li-rich cathode, DNP-NMR, interfacial phenomena, anode-cathode crosstalk

## Abstract

Performance decline in Li-excess cathodes is generally attributed to structural degradation at the electrode-electrolyte interphase, including transition metal migration into the lithium layer and oxygen evolution into the electrolyte. Reactions between these new surface structures and/or reactive oxygen species in the electrolyte can lead to the formation of a cathode electrolyte interphase (CEI) on the surface of the electrode, though the link between CEI composition and the performance of Li-excess materials is not well understood. To bridge this gap in understanding, we use solid-state nuclear magnetic resonance (SSNMR) spectroscopy, dynamic nuclear polarization (DNP) NMR, and electrochemical impedance spectroscopy (EIS) to assess the chemical composition and impedance of the CEI on Li_2_RuO_3_ as a function of state of charge and cycle number. We show that the CEI that forms on Li_2_RuO_3_ when cycled in carbonate-containing electrolytes is similar to the solid electrolyte interphase (SEI) that has been observed on anode materials, containing components such as PEO, Li acetate, carbonates, and LiF. The CEI composition deposited on the cathode surface on charge is chemically distinct from that observed upon discharge, supporting the notion of crosstalk between the SEI and the CEI, with Li^+^-coordinating species leaving the CEI during delithiation. Migration of the outer CEI combined with the accumulation of poor ionic conducting components on the static inner CEI may contribute to the loss of performance over time in Li-excess cathode materials.

## Introduction

The next generation of high energy density lithium ion batteries will likely be defined by the choice of cathode (Goodenough and Kim, 2009; Ellis et al., 2010; Etacheri et al., 2011). Thus, current research has focused on identifying cathode materials that can provide both high capacity and high voltage to pair with existing anode materials. Li-excess transition metal oxide cathodes are promising candidates, as they offer high capacities exceeding 250 mAh/g (Sathiya et al., 2013; Rozier and Tarascon, 2015; Arunkumar et al., 2016; Hy et al., 2016), which is a substantial improvement over commercial cathodes LiNi_0.8_Co_0.15_Al_0.05_O_2_ (NCA) and LiNi_x_Mn_y_Co_z_O_2_ (NMC) at ~200 mAh/g (Hy et al., 2016). In Li-excess compounds, Li is substituted in the transition metal layer allowing for a combination of transition metal (TM) and anion (e.g., oxygen) redox to contribute to the observed capacity (Zheng et al., 2019). However, structural degradation during the repeated delithiation/lithiation that occurs during electrochemical cycling causes severe capacity and voltage fade, which ultimately hinders the practical use of these materials in commercial lithium ion batteries (Hy et al., 2016).

The capacity and voltage fading in Li-excess cathodes has mainly been attributed to a combination of TM migration and oxygen loss from the lattice (Song et al., 2012; Mohanty et al., 2014; Sathiya et al., 2015; Hy et al., 2016; Jung et al., 2017). Oxygen evolution in these systems has been proposed to lead to a nucleophilic attack of the carbonate electrolyte (Aurbach et al., 1991; Yabuuchi et al., 2011; Dupré et al., 2015; Gauthier et al., 2015). Electrolyte decomposition products can then go on to form a cathode electrolyte interphase (CEI) (Aurbach et al., 1991) that is also correlated with irreversible capacity loss in the first cycle (Zhang et al., 2002a). While a substantial body of work has examined the relationship between structural rearrangements in the electrode as a function of electrochemical cycling, little is known about the role that the CEI plays in the performance decline of Li-excess cathodes.

Previous characterizations of the CEI suggest that its composition is nearly identical to that of the solid electrolyte interphase (SEI) on the anode side of the battery. For example, Fourier transform infrared (FTIR) spectroscopy (Aurbach et al., 1991; Kanamura et al., 1996; Aurbach, 1998; Hong et al., 2012; Zhang et al., 2020), mass spectrometry (MS) (Liu et al., 2016), and X-ray spectroscopies (Eriksson et al., 2002; Edström et al., 2004; Lu et al., 2009; Carroll et al., 2013; Malmgren et al., 2013; Yamamoto et al., 2014; Jarry et al., 2015; Fang et al., 2018; Källquist et al., 2019; Li et al., 2019) show that the CEI contains commonly observed electrolyte decomposition products such as PEO-type polymers, organic and inorganic carbonates, inorganic oxides, and fluorinated compounds. The similarity in composition between the CEI and the anodic SEI has led to the speculation that the CEI is formed from anode-cathode crosstalk (Cuisinier et al., 2013; Lu et al., 2013; Malmgren et al., 2013; Fang et al., 2018). However, a solely anode-derived CEI ignores the contribution of cathode-specific degradation mechanisms (e.g., oxygen evolution) and surface chemistries (e.g., undercoordinated transition metal centers) (Zhang et al., 2002a; Castaing et al., 2015) to electrolyte decomposition and subsequent interphase formation. Techniques that identify how molecular-level structures within the CEI evolve during battery operation are required to understand the relationship between CEI growth, anode-cathode crosstalk, and performance degradation.

Here, we use a combination of solid-state NMR (SSNMR), dynamic nuclear polarization (DNP) NMR, and electrochemical impedance spectroscopy (EIS) measurements to probe the organic and inorganic CEI that forms on Li_2_RuO_3_ cathodes at different states of charge. While both SSNMR and DNP NMR have enabled the assignment of the SEI on graphitic and Si anodes (Leifer et al., 2011; Michan et al., 2016; Leskes et al., 2017; Jin et al., 2018), reports examining the cathode side of the battery have been more limited (Cuisinier et al., 2012, 2013) because most commercially available cathode materials are strongly paramagnetic (Dupre et al., 2011). In this report, SSNMR/DNP characterization is enabled by the reduced magnetic susceptibility in Li_2_RuO_3_ (Miura et al., 2007; Wang et al., 2014), which allows us to identify the composition and the structure of the CEI. We find that the CEI on Li_2_RuO_3_ is comprised of a mixture of organic and inorganic components, such as PEO, Li acetate, carbonates, and LiF that arise from the decomposition of carbonate solvents and LiPF_6_. Formation of the CEI is observed after the first charge of the battery, indicating that cathode degradation during delithiation can lead to electrolyte decomposition. The CEI that is observed in the charged state is distinct from that observed in the discharged state, supporting the hypothesis that crosstalk between the SEI and the CEI impacts the compositions of these interphases, where solvating components leave the CEI during delithiation. Migration of the outer CEI combined with the accumulation of poorly ionic conducting components on the static inner CEI may contribute to the loss of performance over time in Li-rich cathode materials.

## Materials and Methods

### Materials

Li_2_CO_3_ (99.99%, trace metals basis), 1 M LiPF_6_ in ethylene carbonate:dimethyl carbonate (EC:DMC 1:1 v/v, LP30, battery grade), fluoroethylene carbonate (FEC, 99%), 1-methyl-2-pyrrolidinone (NMP, anhydrous, 99.5%), 1,1,2,2-tetrachloroethane (TCE, ≥98.0%), and KBr (≥99.0%) were purchased from Sigma-Aldrich. Prior to use, FEC was dried over molecular sieves in an Ar-filled glovebox (O_2_ <0.1 ppm, H_2_O <0.5 ppm) for 48 h. KBr was dried in vacuo at 100°C for 3 days before bringing into the glovebox for use. All other chemicals were used as received. RuO_2_ (99.95%, trace metals basis) was purchased from Alfa Aesar and dried at 300°C for 4 h to remove moisture prior to syntheses. Carbon Super P and polyvinyldene fluoride (PVDF) were purchased from MTI Corporation and used as received. TEKPol was purchased from CortecNet; CDCl_3_ was purchased from Cambridge Isotope Laboratories, both were used as received.

### Synthesis of Li_2_RuO_3_

Li_2_RuO_3_ was prepared by traditional solid-state synthesis by reacting Li_2_CO_3_ with RuO_2_. Stoichiometric amounts of Li_2_CO_3_ (10% wt excess) and dried RuO_2_ powders (vide supra) were combined and hand ground in a mortar and pestle for 10 min. The mixture was heated in an alumina crucible at 900°C for 12 h and then at 1,000°C for 12 h using a 2°C min^−1^ ramp rate with intermediate and final grinding for 10 min. Synthesis of Li_2_RuO_3_ was confirmed using powder X-ray diffraction (PXRD) (Figure S1).

### Powder X-Ray Diffraction

PXRD was collected on a PANalytical XPERT3 powder diffractometer with Cu Kα radiation. The Li_2_RuO_3_ sample was placed on a zero-background Si plate for data collection.

### Electrode Fabrication

The Li_2_RuO_3_ cathodes were prepared by first grinding a 9:1 ratio by mass mixture of Li_2_RuO_3_ and carbon super P. This mixture was added to a solution of PVDF binder in a 9:1 ratio by mass (Li_2_RuO_3_ + C:PVDF), using NMP as the PVDF solvent, to create a viscous slurry. The slurry was cast onto an Al current collector (25 μm thick) using a 150 μm doctor blade and dried at 100°C in a vacuum oven overnight. The dried film was punched into 12.7 mm diameter disks to use in cell assembly. Typical mass loadings of active material (Li_2_RuO_3_) per cathode were 7–12 mg cm^−2^. These electrodes were used for all electrochemical testing and NMR characterization.

### Electrochemistry

Electrochemical tests were conducted using 2032 coin cells assembled in an Ar-filled glovebox with a Li-metal disc as the anode and Li_2_RuO_3_ composite thin films as the cathode. Each assembled cell used Whatman glass microfiber (GF/A) separators and ~0.2 mL battery grade LP30 or LP30 + 10% FEC v/v electrolyte. Galvanostatic cycling experiments were performed at rates of C/30 to C/10 (assuming a theoretical capacity of 329 mAh g^−1^) between 2.0 and 4.6 V.

### DNP NMR

DNP NMR experiments were performed at 9.4 T at Bruker Biospin in Billerica, MA on a 400 MHz Avance III HD spectrometer with a 263.6 GHz gyrotron microwave source using a 3.2 mm HXY MAS probehead. DNP NMR experiments at 14.1 T were performed at the New York Structural Biology Center (NYSBC) on a 600 MHz Avance III spectrometer with a 395 GHz gyrotron microwave source using a 3.2 mm HCN MAS probehead. Cells were disassembled in either the charged or discharged state at 4.6 or 2.0 V, respectively. After cell disassembly, the Li_2_RuO_3_ cathode material was scraped from the Al current collector and was then dried under vacuum for 1 h to remove residual EC/DMC. Note electrodes were not washed during sample preparation in order to preserve the highly sensitive interphase layer. For analysis in Billerica, samples were sealed under Ar and transported to the Bruker Biospin facility in three layers of sealed plastic bags. Upon arrival, the samples were packed into 3.2 mm sapphire MAS rotors in a N_2_-filled glovebox. For measurements at NYSBC, samples were packed in an Ar glovebox. Each sample was mixed with KBr powder in a roughly 1:1 ratio by volume in a mortar and pestle until homogenized. 10–20 μL of radical solution (20 mM TEKPol in 4:1 TCE:CDCl_3_ solution) was added to the sample and ground until fully wetted. Samples were immediately packed into 3.2 mm sapphire rotors. Si spacers were used to protect the samples from moisture and oxygen. Electron paramagnetic resonance (EPR) spectroscopy was recorded on a Bruker EMXnano benchtop EPR spectrometer to measure the concentration of TEKPol in the solution after integration with the sample (Figure S2). Sample masses, volumes of radical solution used, and EPR results are available in Table S1. All experiments were performed at 11.1 kHz magic-angle spinning (MAS) frequency, except for the 1 cycle LP30 sample which was spun at 10 kHz. DNP NMR was recorded at probe sensor temperatures of 90-112 K. ^1^H → ^13^C cross polarization magic-angle spinning (CPMAS) DNP NMR experiments were performed using ^13^C *B*_1_ field of ~60 kHz and ^1^H field linearly ramped from 80 to 100% at the Hartmann-Hahn matching condition followed by 100 kHz SPINAL-64 decoupling on ^1^H. ^1^H → ^13^C CPMAS spectra were recorded with microwaves on and microwaves off to calculate signal enhancements from DNP. At 9.4 T, we find that ε ~38 (Figure S3), whereas ε ~6 at 14.1 T.

### Solid-State NMR

SSNMR experiments were performed at room temperature on a Bruker Avance NEO 600 MHz spectrometer equipped with a 1.6 mm HFXY MAS Phoenix probehead. The cathode material was extracted and dried as described in the previous section, either in the discharged state at 2.0 V or the charged state at 4.6 V. Each sample was mixed with KBr powder in a roughly 1:1 ratio by volume in a mortar and pestle until homogenized, then packed into a 1.6 mm o.d. ZrO_2_ rotor in an Ar-filled glovebox. All experiments were performed at 18 kHz MAS frequency. ^1^H → ^13^C CPMAS and ^19^F → ^13^C CPMAS experiments were performed using ^13^C *B*_1_ field of ~60 kHz and ^1^H (or ^19^F, respectively) field linearly ramped from 90 to 100% at the Hartmann-Hahn matching condition of ~60 kHz and high power ^1^H decoupling with TPPM for both experiments. ^19^F spin echo spectra were collected using a rotor-synchronized spin-echo pulse sequence (90°—τ—180°—τ—acquire, with τ set to 2 rotor periods).

## Results and Discussion

### Capacity and Voltage Fade During Galvanostatic Cycling of Li_2_RuO_3_

**Figure 1:**
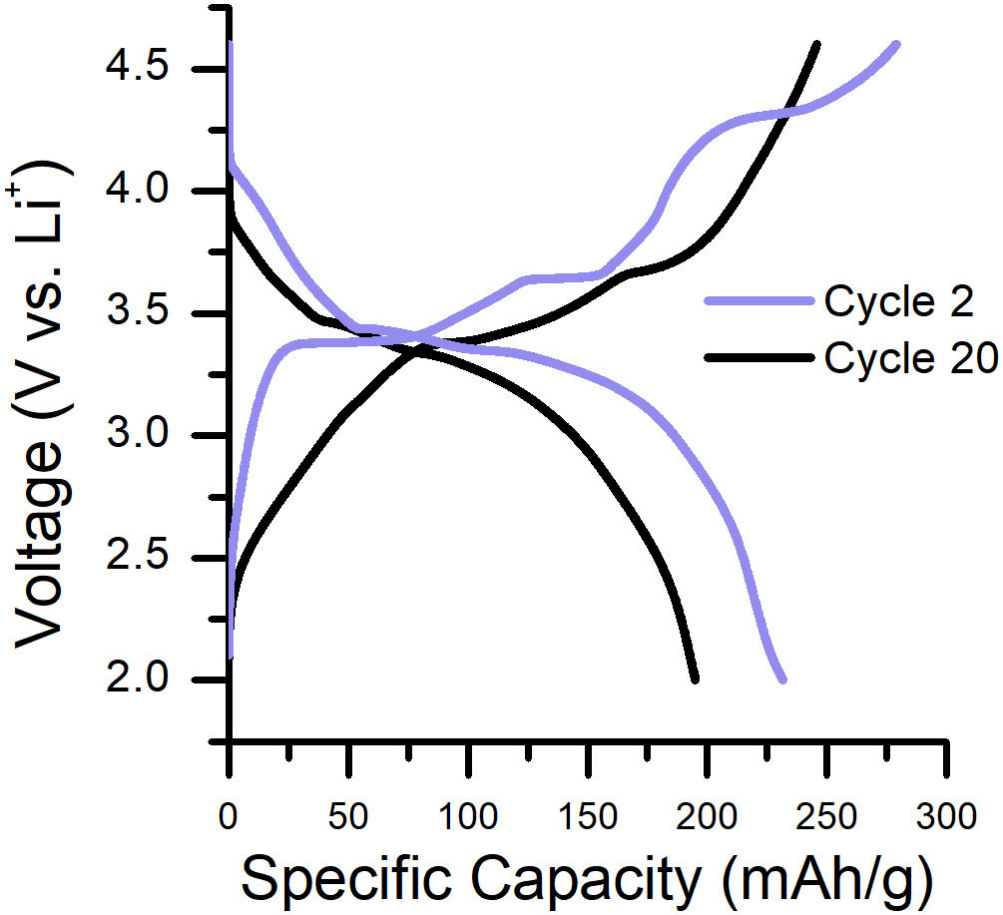
Characteristic charge/discharge curves for a Li_2_RuO_3_|Li cell cycled in LP30 at C/30. Cycle 2 and cycle 20 are shown to emphasize the capacity/voltage fade that occurs during cycling. Specific capacity as a function of cycle number is shown in Figure S4.

Characteristic charge/discharge plots for the 2nd and 20th cycles of Li_2_RuO_3_|Li batteries cycled in LP30 are shown in Figure 1. The delithiation/lithiation behavior of Li_2_RuO_3_ has been studied extensively to determine structure-performance relationships in Li-excess cathodes (e.g., the role of TM migration and oxygen loss) because it contains a simple redox couple (where Ru^4+^ is oxidized to Ru^5+^ upon charge) (Sathiya et al., 2013; Mori et al., 2016; Zheng et al., 2019). At early charge cycles (Figure 1, purple line), two prominent plateaus are observed at 3.42 and 3.65 V, representing two separate phase transformations that occur during removal of the first lithium (Li_2−*x*_RuO_3_ where *x* = 1) (Zheng et al., 2019). By the end of the second plateau, ex situ diffraction, X-ray scattering, and density functional theory (DFT) indicate that the *C*2*/c* crystallographic structure rearranges into a *R*$\bar{3}$ intermediate phase for LiRuO_3_ (Sathiya et al., 2013, 2015; Mori et al., 2016; Zheng et al., 2019). Upon further charging, a third sloped plateau is observed at 4.30 V which signifies local reordering of the *R*$\bar{3}$ intermediate phase to a Li-deficient rhombohedral phase (Mori et al., 2016; Zheng et al., 2019). Beyond 4.30 V, Ru migration and oxygen redox/evolution may occur to compensate charge on the now undercoordinated oxygen (Sathiya et al., 2013; Mori et al., 2016; Zheng et al., 2019). The structural transitions that follow in the discharge process are distinct from that of the charge process; the plateau at approximately 3.30 V represents the reduction of Ru^5+^ to Ru^4+^ and corresponds to a single-phase transition between the reordered Li_2_RuO_3_ (*C*2*/c*, post-TM migration) and LiRuO_3_ (*R*$\bar{3}$, post-TM migration) (Mori et al., 2016; Zheng et al., 2019). Upon subsequent charge/discharge, the voltage plateaus subside such that the shape of the voltage profile is more consistent cycle-to-cycle, indicating lithium insertion/removal is reversible in these structures (Sathiya et al., 2013; Zheng et al., 2019). By the 20th cycle, voltage fade and capacity decline are evident (Figure 1, black line) and continue to worsen with cycle number (Figure S4). This gradual performance loss is attributed to the accumulation of Ru cations in the Li layers of the cathode (Sathiya et al., 2015; Zheng et al., 2019) and/or accumulation of electrolyte decomposition products on the cathode surface (Zhang et al., 2002b, 2006; Gauthier et al., 2015).

### NMR Characterization of the CEI Composition

**Figure 2:**
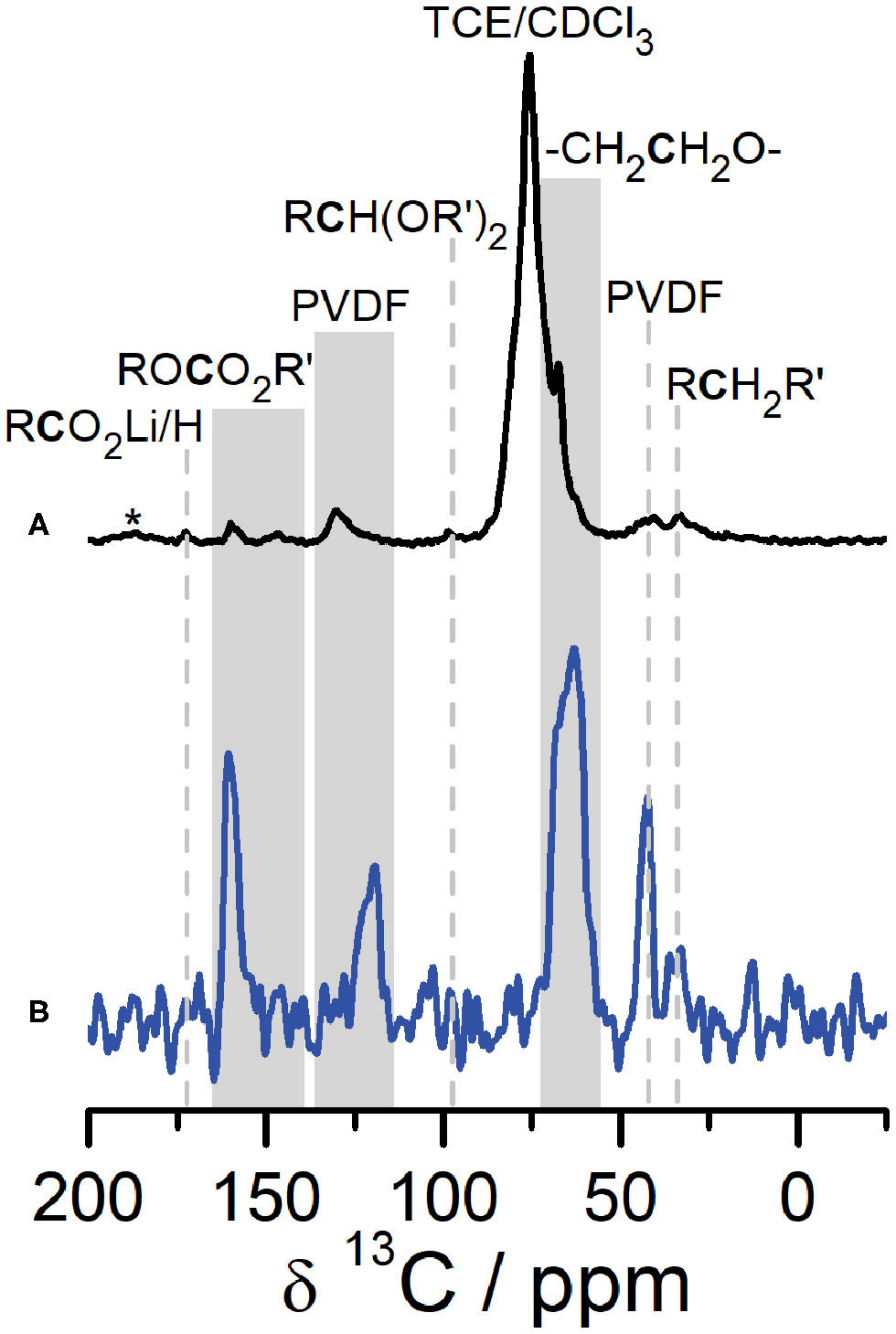
^1^H→^13^C CPMAS DNP NMR (**A**, black) and SSNMR (**B**, blue) of the CEI on Li_2_RuO_3_ cycled against Li in LP30 at C/10 and disassembled at the end of the 27th cycle (at discharge, 2.0 V). SSNMR (blue) was collected at 14.1 T at room temperature. DNP NMR (black) was recorded at 9.4 T at 92 K. Gray shading is used to label peaks in the ROCO_2_R′ region as well as to label regions where the temperature change caused slight shifts in peak centers between the DNP and SSNMR spectra. *denotes spinning sidebands.

A combination of SSNMR and DNP NMR was used to identify the structure and composition of the CEI that forms on Li_2_RuO_3_ cathodes. Figure 2 shows a comparison of ^1^H → ^13^C CPMAS NMR of the CEI on Li_2_RuO_3_ in the discharged state (at 2.0 V) after 27 cycles collected under DNP conditions (Figure 2A, black) and conventional SSNMR (Figure 2B, blue). The most striking feature from these spectra is that the signal-to-noise ratio (SNR) in the DNP NMR is significantly improved compared to conventional SSNMR as a result of the DNP enhancement for surface species (ε ~38 at 9.4 T, Figure S3). Both spectra show nearly identical resonances (N.B. small changes in shift between the two spectra may arise because DNP NMR is performed at cryogenic temperatures) with DNP NMR showing minor peaks at 98, 148, and 173 ppm not observed in conventional SSNMR due to the lower SNR.

Analysis of the ^1^H → ^13^C CPMAS NMR spectra indicates that the CEI on Li_2_RuO_3_ contains several electrolyte decomposition products commonly found in the SEI. The ^13^C resonances in the carbonyl region can be assigned to Li acetate at 173 ppm and Li alkyl carbonates/polycarbonates at 160 ppm (Leskes et al., 2017). The ^13^C resonance at ~68 ppm is consistent with PEO-type fragments in the CEI, which is also a prominent component of the SEI on the anode side of the battery (Edström et al., 2004; Gireaud et al., 2006; Leskes et al., 2017; Jin et al., 2018). The low intensity ^13^C peak at 148 ppm is assigned to alkyl carbonate environments (e.g., RO*C*O_2_R′) formed from carbonate solvent decomposition (Schechter et al., 1999; Dedryvère et al., 2005; Leifer et al., 2011; Michan et al., 2016). The ^13^C resonance observed at approximately 98 ppm is assigned to an acetal carbon (R*C*H(OR′)_2_) moiety, prominently observed on the anode in the presence of FEC additive (Jin et al., 2018). Here, acetal decomposition products are observed in the CEI on Li_2_RuO_3_ both with and without FEC (Figures S10–S12). Acetal carbons have previously been attributed to the reductive decomposition of the carbonyl in carbonate-based electrolytes and additives (Leifer et al., 2011; Michan et al., 2016; Jin et al., 2018) and we hypothesize that they migrate to the cathode side of the battery during discharge (vide infra). The ^13^C NMR also shows two additional resonances at 121 ppm and 43 ppm, not usually observed in binder-free characterization of the SEI, that we assign to the PVDF in the cathode composite (see Figures S5–S7 for further discussion on this assignment).

**Figure 3:**
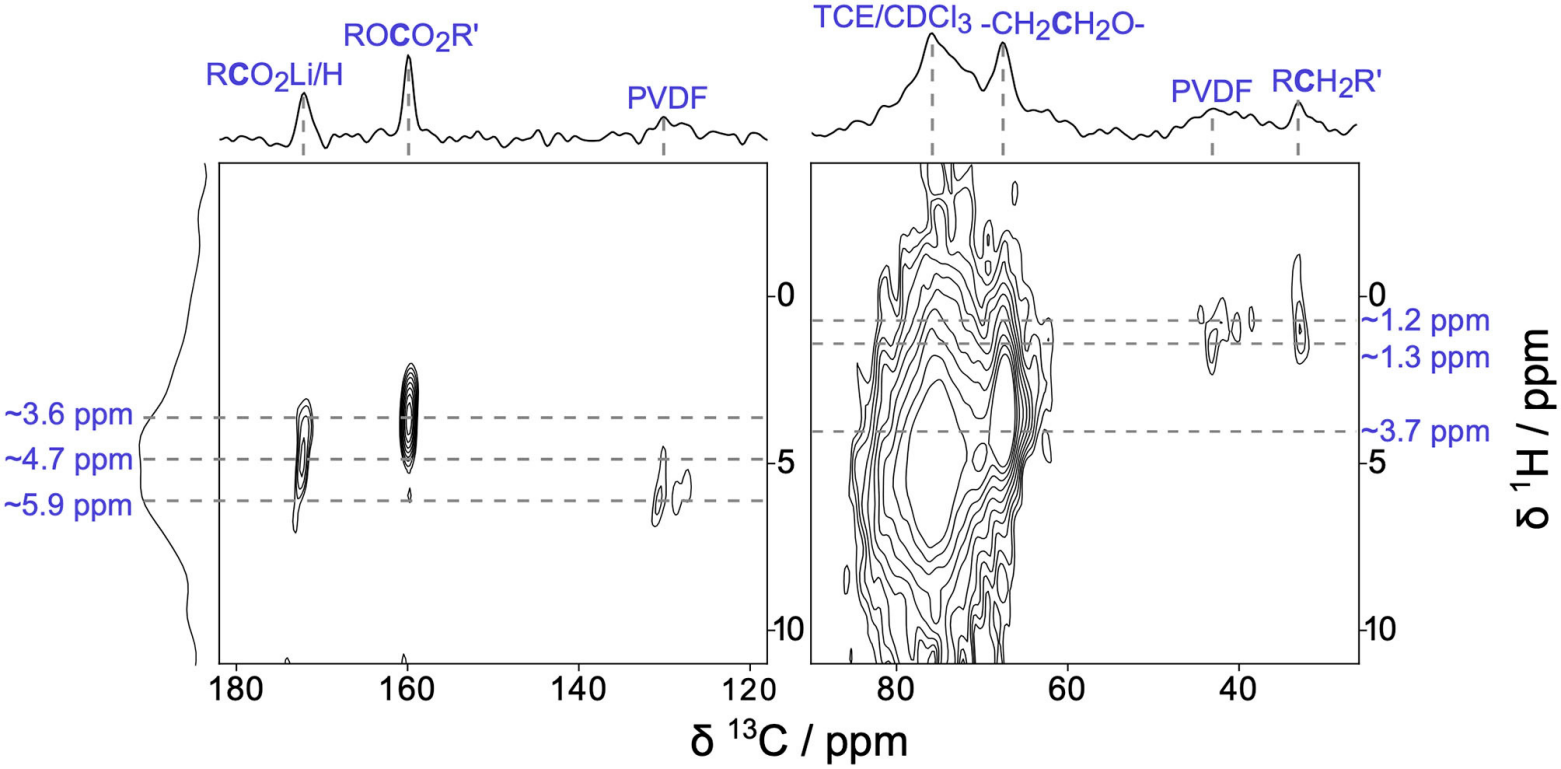
Two-dimensional (2D) ^1^H→^13^C heteronuclear correlation (HETCOR) DNP NMR of Li_2_RuO_3_ cycled against Li in LP30 at C/10 and disassembled at the end of the 1st charge/discharge cycle at 2.0 V.

To further explore the surface enhancement provided by DNP, we examined the CEI present on Li_2_RuO_3_ cathodes after a single charge/discharge cycle with two-dimensional (2D) ^1^H → ^13^C heteronuclear correlation (HETCOR) DNP NMR (Figure 3). Figure 3 shows the remarkable sensitivity and in-depth structural assignments that can be obtained from 2D DNP NMR analyses of Li_2_RuO_3_ cathode materials when the CEI is still expected to be very thin (a few nanometers). The ^1^H → ^13^C HETCOR results confirm our above assignments of PEO, Li acetate, and carbonates in the CEI. The ^13^C resonance at ~68 ppm has a corresponding ^1^H crosspeak at 3.68 ppm, which is consistent with the -(CH_2_*C*H_2_O)_*n*_- fragments found in PEO (Michan et al., 2016; Leskes et al., 2017). Both the alkyl carbonates and lithium acetate species show characteristic correlations that we would expect for these individual species (^13^C ~160 ppm, ^1^H ~3.62 ppm and ^13^C ~173 ppm, ^1^H ~4.67 ppm, respectively) (Michan et al., 2016; Jin et al., 2017; Leskes et al., 2017). The peaks at 98 ppm and 148 ppm seen in Figure 2 are not visible in the HETCOR due to the low SNR in those regions. In the aliphatic portion of the spectrum (~30–50 ppm), we see two cross peaks between ^13^C and ^1^H. The ^13^C resonance at 32 ppm exhibits a ^1^H crosspeak at 1.21 ppm and can be assigned to a R*C*H_2_R'-type species, such as lithium butylene dicarbonate (LBDC) (Michan et al., 2016). The crosspeak for ^13^C at 43 ppm and ^1^H at 1.34 ppm is consistent with R*C*F_x_ groups in PVDF binder (Montina et al., 2012). Further scrutiny of the ^1^H → ^13^C HETCOR data reveals that the ^13^C resonance at ~121 ppm shows a ^1^H crosspeak with a center of mass at ~5.87 ppm, which is consistent with the hydrocarbons adjacent to CF_x_ moeities in PVDF (Montina et al., 2012). Overall, this HETCOR spectrum confirms that after just one cycle, a multicomponent CEI forms containing PEO-type polymers, alkyl carbonates, and Li salts.

Similar to the organic CEI, the inorganic CEI contains electrolyte decomposition products that have previously been observed on the anode side of the battery. Figure S7 shows ^19^F SSNMR of Li_2_RuO_3_ after electrochemical cycling in LP30. The most intense ^19^F resonances are a doublet at −73 ppm that is assigned to residual ${\text{PF}}_{6}^{-}$ salt and a ^19^F resonance at −203 ppm that is assigned to LiF that arises from the decomposition of LiPF_6_.

### CEI Dependence on State of Charge and CEI/SEI Crosstalk

**Figure 4:**
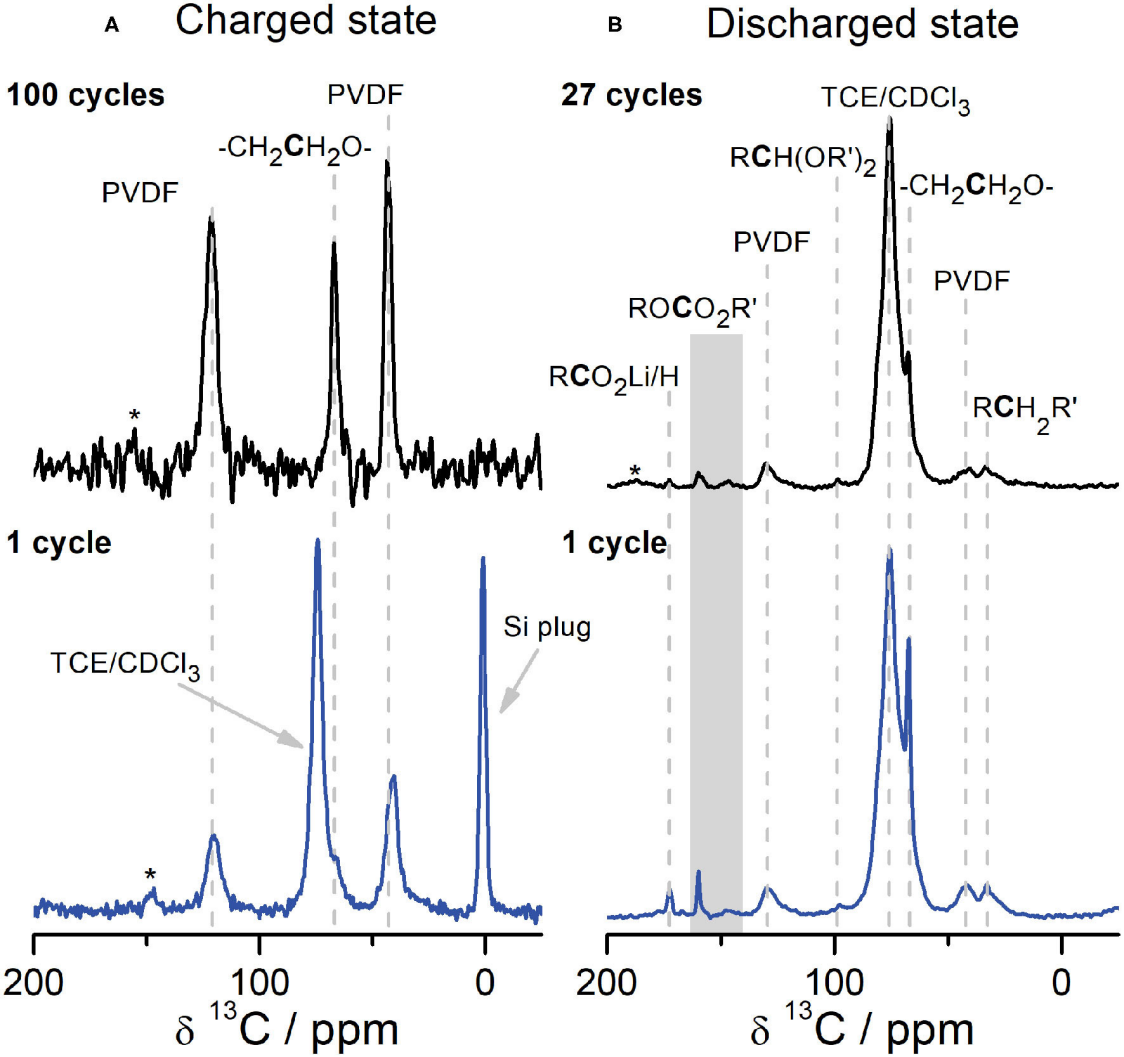
^1^H→^13^C CPMAS SSNMR and DNP NMR spectra of the CEI on Li_2_RuO_3_ at different states of charge and cycle number. The cells shown in **(A)** were disassembled in the charged state at 4.6 V after a single charge (blue, bottom left, DNP NMR at 14.1 T at 100 K) and 100 cycles (black, top left, SSNMR at 14.1 T, room temperature). The cells shown in **(B)** were disassembled in the discharged state at 2.0 V after one charge/discharge cycle (blue, bottom right, DNP NMR at 9.4 T at 92 K) and 27 cycles (black, top right, DNP NMR at 9.4 T at 92 K). All samples were cycled in LP30 against Li metal at a rate of C/10. The gray rectangle in **(B)** is used to label peaks at 148 ppm and 160 ppm in the ROCO_2_R′ region. *denotes spinning sidebands.

To understand the formation of the CEI, we performed NMR analyses as a function of state of charge and cycle number (Figure 4); we will discuss the latter first. When comparing the molecular species that are present in the CEI after the first cycle and many cycles (27 cycles for cells disassembled at 2.0 V and 100 cycles for cells disassembled at 4.6 V), we see that the chemical composition of the CEI remains relatively constant for a given state of charge (i.e., the species present within Figure 4A are similar to one another; likewise for Figure 4B). The presence of PVDF on the surface of Li_2_RuO_3_ cathodes allows us to perform a semi-quantitative analysis of the voltage-dependence of the CEI composition by comparing the relative intensities in Figure 4. For example, after the first charge cycle (Figure 4A, bottom), a small shoulder next to the solvent peak at ~68 ppm indicates that PEO from EC decomposition deposits on the cathode surface. Peak fitting of this spectrum shows a ratio of PEO:PVDF equal to 0.28 (see Table S2 for tabulated peak integrals). After 100 cycles, this PEO resonance persists and is present in similar quantities with a ratio of PEO:PVDF equal to 0.29. Similarly, the PEO:PVDF ratio in the discharged state is consistent across cycle number: approximately 2.2 after 1 cycle and 2.0 after 27 cycles. Resonances assigned to Li alkyl carbonates (148 and 160 ppm), LBDC (32 ppm), cross-linking acetal carbons (98 ppm), and Li acetate (173 ppm) are consistently observed in cells disassembled at 2.0 V from the first cycle onward (Figure 4B). The buildup of CEI products on the first charge/discharge cycle at high operating potentials is consistent with work from Komaba and coworkers that show O_2_ released upon charge from cathode degradation can be reduced to reactive oxygen species, e.g., O^2−^, around 3.0 V (Yabuuchi et al., 2011). On discharge, these species can react with the carbonate electrolyte and deposit on the cathode surface (Yabuuchi et al., 2011), indicating that cathode degradation is at least partly responsible for interphase formation.

The differences observed in CEI composition between charge and discharge suggest that a substantial amount of the CEI is stripped off during charge, likely due to anode-cathode crosstalk. For example, the PEO:PVDF ratio in the discharged state significantly decreases from ~2 in the discharged state to ~0.29 in the charged state. Similarly, Li acetate, acetal carbon moieties, and Li carbonate species are absent in the charged state (Figure 4A), although they were clearly present at the end of discharge (Figure 4B). The lack of Li salts and some PEO in the CEI on charge indicates that upon delithiation, these CEI compounds solvate Li ions when traveling to the anode side of the battery. These species are subsequently re-deposited on the cathode surface during the next discharge step (Figure 4B). The phenomenon of migration and crosstalk between the anode and the cathode has been shown by several groups (Cuisinier et al., 2013; Zhan et al., 2013; Fang et al., 2018; Harris et al., 2018; Källquist et al., 2019). For example, Hamers and coworkers observed migration of species from the SEI on graphite to the CEI on NMC during cycling (Fang et al., 2018). Yet no evidence of CEI formation on NMC was observed when cycled against higher voltage anodes, such as Li titanate (LTO), supporting the hypothesis that many SEI species only form at lower voltages (Fang et al., 2018). The lack of CEI in NMC|LTO cells led to the hypothesis that CEI formation may rely on electrolyte degradation at low voltage (because very little would occur in LTO-containing cells) and anode-cathode crosstalk, rather than cathode degradation-driven processes that can occur at higher voltages. However, analysis of Li_2_RuO_3_ cathodes after just a single charge to 4.6 V show the deposition of PEO (Figure 4A), suggesting that the structural rearrangements that occur in less stable Li-rich cathode materials may lead to unique CEI formation mechanisms that act in parallel with anode-cathode crosstalk. In support of cathode degradation contributing to the CEI, Dupré and coworkers studying LiFePO_4_|LTO cells found that a composite inorganic/organic CEI formed despite cycling against these “SEI-free” anodes (Castaing et al., 2015). Further, the stripping and deposition of the CEI that we observe during delithiation/lithiation is consistent with XPS measurements by Hahlin and coworkers, where they observe CEI thickening upon discharge of Li_2_VO_2_F cathodes (Källquist et al., 2019). They suggest this CEI thickening at discharge is correlated with phase changes at the surface of the electrode that migrate to the electrode bulk upon further (de)lithiation (Källquist et al., 2019). The coupling of bulk electrode degradation and interfacial instability was correlated with a continual loss of capacity during electrochemical cycling (Källquist et al., 2019), and is likely partially responsible for the capacity fade observed in Li_2_RuO_3_ as well.

Upon extended cycling of Li_2_RuO_3_, the capacity gradually fades (i.e., Figure S13C shows a discharge capacity loss of 87% after 100 cycles). EIS results (Table S3, Figures S8, S9) show that the interfacial resistance (R_CEI_) increases when measured in the discharged state (2.0 V) compared to the charged state (4.6 V), which is correlated with the CEI growth that we detect with NMR spectroscopy (Figure 4). This phenomenon has been documented in other EIS work on cathode materials (Zhang et al., 2002a; Nagao et al., 2019) and attributed to CEI dissolution at high voltages/deposition at low voltages (Zhang et al., 2002b). Our NMR analyses of Li_2_RuO_3_ cathodes provides molecular-scale evidence supporting this theory. More specifically, our results suggest there may be an increase in interfacial resistance at the discharged state due to deposition of Li salts/short chain PEO at low voltages. In addition, we also observe that certain species, like PEO, persist in the CEI from the first charge onwards, regardless of cycle number or state-of-charge. Similarly, LiF is present on the cathode in both the charged and discharged state and does not appear to depend greatly on state of charge (Figure S7). We hypothesize that the PEO observed on the cathode surface during charge is likely longer-chain PEO compounds, which could explain why some PEO (likely short-chain) is removed during charge but not all. The crystallinity, quantity, and arrangement of PEO and Li salts on the surface of Li_2_RuO_3_, in addition to the charge-dependent CEI stripping/deposition, may be correlated with cycling performance of Li ion batteries. Crosstalk between electrode interphases may negatively impact battery performance by promoting thickening of the anodic SEI over extended cycling, which leads to reduced ion transport at the negative electrode (Burns et al., 2013). It remains unclear if the CEI of this material is deposited evenly such that it acts as a passivating layer or if the CEI deposits unevenly on the surface, welcoming parasitic attacks (Cuisinier et al., 2013). An uneven or fluxional CEI may leave the electrode surface vulnerable to opportunistic degradation of the cathode surface, such as HF reacting with the surface to form insulating species like LiF, and ultimately lead to performance degradation (Hong et al., 2012).

## Conclusions

The high surface sensitivity and chemical resolution afforded by DNP and SSNMR techniques have allowed structural assignment of the chemical species (e.g., PEO, Li salts, carbonates, and LiF) present in the CEI on Li_2_RuO_3_ cathodes after a single charge/discharge cycle and at different stages of the charge/discharge process. These findings provide the framework necessary to correlate well-documented voltage-dependent cathode degradation processes to specific molecular compounds in both the anode- and cathode-electrolyte interphases. Reversible deposition and stripping of Li salts/short-chain PEO in the CEI during anode-cathode crosstalk suggests that these species may play an important role in controlling interfacial resistance and Li^+^ transport through the battery during charge/discharge. Instability in the CEI paired with the accumulation of poor ionic conductors (e.g., LiF and high molecular weight PEO) in the CEI may contribute to performance degradation over time in Li-excess cathode materials. Future work examining the molecular-level evolution of the CEI during electrochemical cycling will play a key role in understanding and controlling Li^+^ ion conduction through this interphase and enabling next generation cathode materials.

## Data Availability Statement

The datasets presented in this study can be found in online repositories. The names of the repository/repositories and accession number(s) can be found below: OSF doi: 10.17605/OSF.IO/NZP82.

## Author Contributions

JH performed materials synthesis, XRD characterization, electrochemistry, and SSNMR experiments. JH, AE, and LM analyzed and interpreted the data and wrote the first draft of the manuscript. MN aided in materials synthesis and electrochemical characterization. IS contributed to the DNP, NMR, and EPR measurements at Bruker Biospin in Billerica, MA. BI contributed to the DNP NMR measurements at NYSBC. JH and LM contributed to the conception and design of the study. The manuscript was edited with input from all co-authors. All authors have read and approved of the submitted version.

## Conflict of Interest

IS was employed by Bruker Biospin Corporation. The remaining authors declare that the research was conducted in the absence of any commercial or financial relationships that could be construed as a potential conflict of interest.
