# Supplementary material for: Reversible Deposition and Stripping of the Cathode Electrolyte Interphase on Li2RuO3
Source: Front Chem. 2020 Aug 4;8:681. doi: 10.3389/fchem.2020.00681 (PMC7417863; doi:10.3389/fchem.2020.00681)
Supplement: Supplementary file 1 [file Data_Sheet_1.pdf]

Supplementary Material

1 XRD

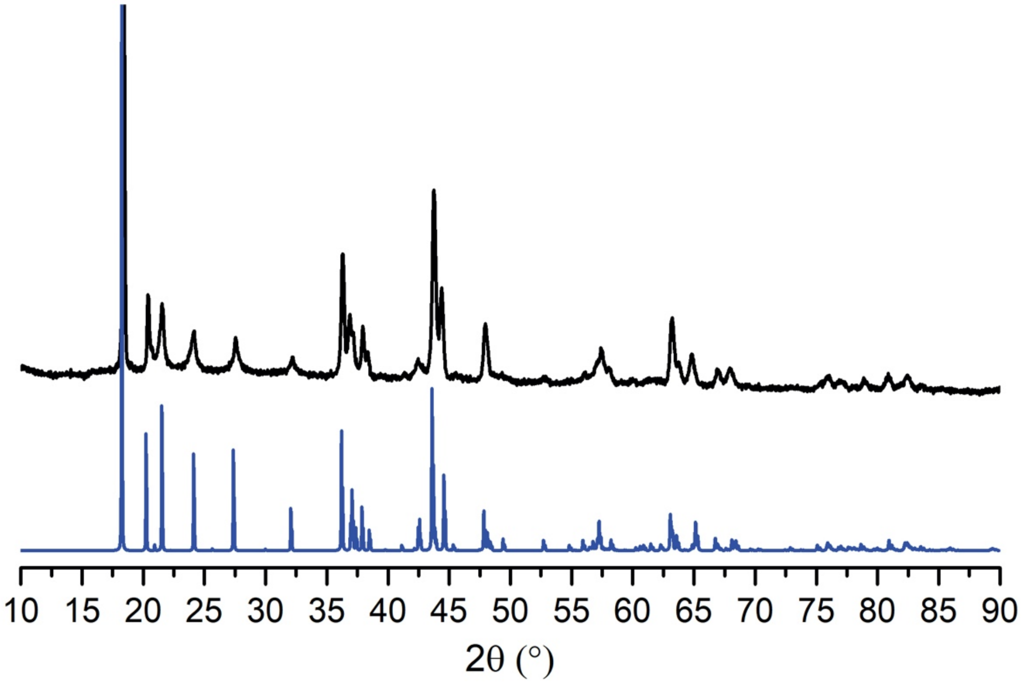

Figure S1: XRD pattern of pristine Li_2RuO_3 powder synthesized by solid-state synthesis (top, black) compared to the calculated pattern for Li_2RuO_3-*C*2/*c* (bottom, blue).

2 Experimental Details of DNP NMR performed at Bruker Biospin (Billerica, MA)

2.1 Electron paramagnetic resonance (EPR) spectrum of Li_2RuO_3 after 27 cycles, disassembled on discharge at 2.0 V

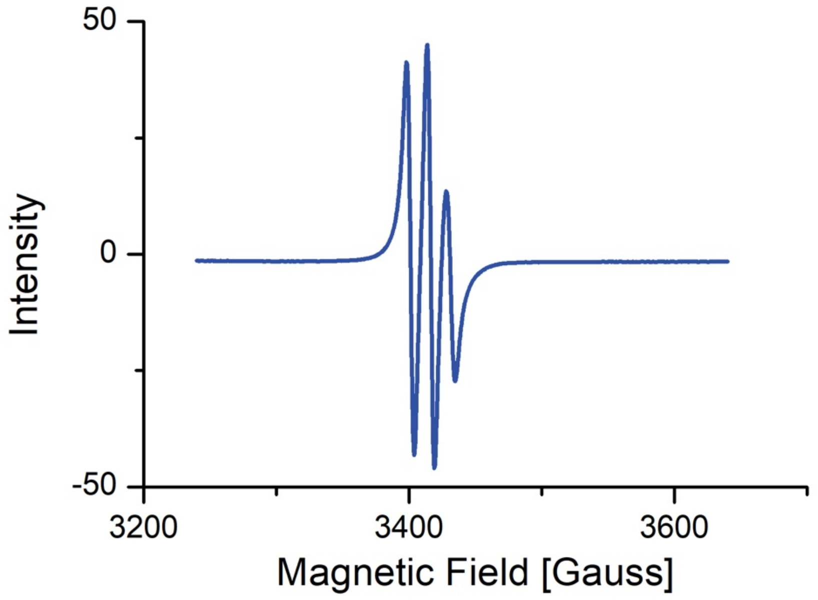

Figure S2: Representative EPR spectrum used to calculate TEKPol concentration in sample for DNP. Individual sample concentrations are listed in Table S1. (Spectrum of Sample 1 from Table S1.)

2.2 Table S1: Sample preparation details

Sample No.	Sample description (disassembled at 2.0 V)	Sample mass (mg)	Active material mass (mg)	Radical solution added (μL)	[TEKPol] in sample (mM)
1	LP30 27 cycles	12.1	5.4	10	2.9
2	LP30 + 10% FEC 27 cycles	9.6	3.3	20	3.8
3	LP30 1 cycle	9.8	3.5	20	5.4

2.3 DNP Enhancement

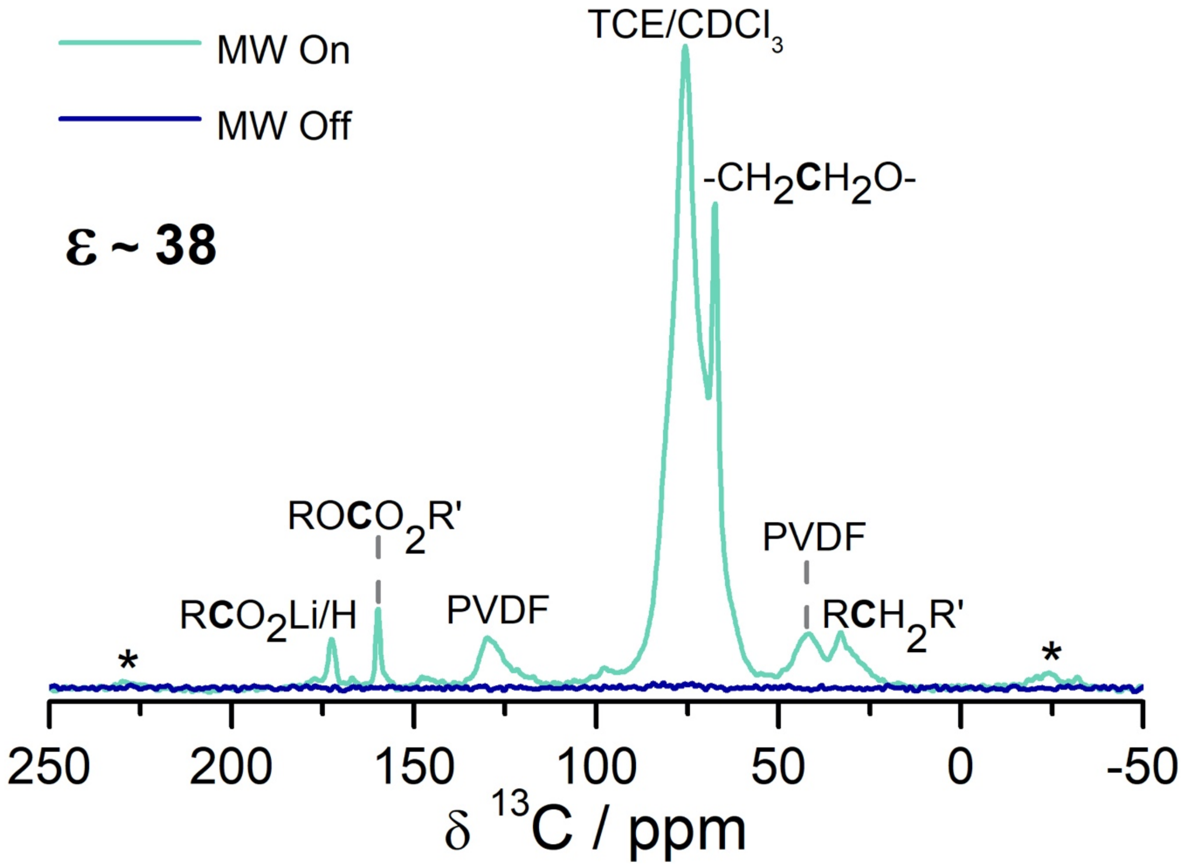

Figure S3: $^1H \rightarrow ^{13}C$ CPMAS DNP NMR spectrum for a Li_2RuO_3 cathode cycled in LP30 against Li metal and disassembled after 27 cycles on discharge recorded at 9.4 T at T = 92 K, with (green) and without (blue) microwave (MW) irradiation. The MW on spectrum shows an enhancement factor, ε, of ~38. This spectrum is the same sample represented in Figure 2a and 4b (top, black).

Note that enhancement in signal appears to be dependent on radical concentration. For example, the improved signal enhancement in the 1 cycle sample in Figure 4b is attributed to higher TEKPol concentration than the 27 cycles sample (5.4 mM vs 2.9 mM from EPR measurements, respectively, Table S1). DNP spectra intensities cannot be compared quantitatively between multiple spectra/different samples.

Table S2: Peak integration of each spectrum shown in Figure 4, which was used to calculate the ratio of PEO to PVDF binder presented in the main text discussion. The TCE/$CDCl_3$ peak at 75 ppm is not included in these calculations of CEI composition.

Peak assignment	Chemical shift center (ppm)	100 cycles, charge<br><br>Peak area fraction (%)	1 cycle, charge<br><br>Peak area fraction (%)	27 cycles, discharge<br><br>Peak area fraction (%)	1 cycle, discharge<br><br>Peak area fraction (%)
**RCH_2R'**	32	0	0	12.4	8.7
**PVDF**	43	32.7	48.0	12.7	10.5
**$-(CH_2CH_2O)_n-$**	68	22.3	21.6	51.6	49.3
**$RCH(OR')_2$**	98	0	0	2.1	5.8
**PVDF**	120-129	45.0	30.4	12.6	11.9
**$ROCO_2R'$**	148	0	0	1.3	3.5
**$ROCO_2R'$**	160	0	0	3.4	5.1
**RCO_2Li/H**	165-173	0	0	3.8	5.2

3 Li_2RuO_3 Electrochemistry

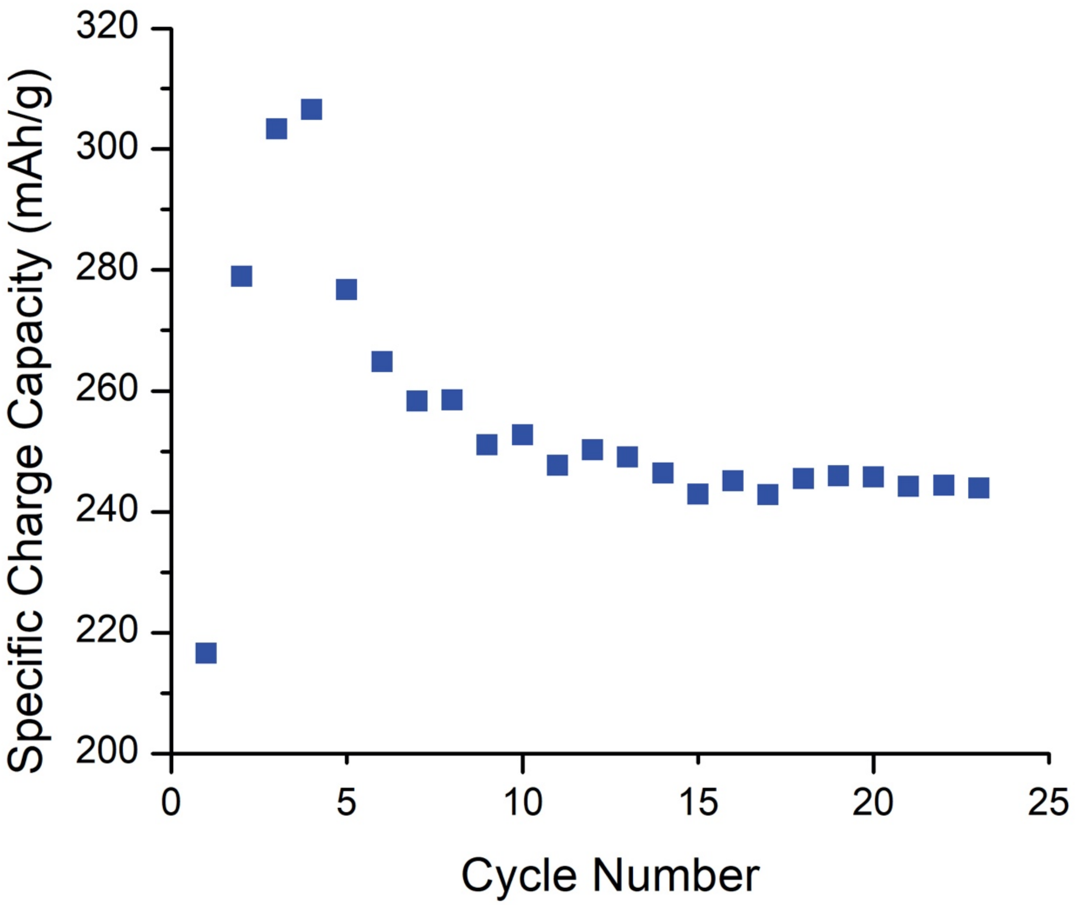

Figure S4: Specific charge capacity for a Li_2RuO_3 cathode cycled in LP30 against Li metal for 23 cycles at a rate of C/30. This sample corresponds to the data shown and analyzed in Figure 1. After first cycle conditioning, high charge capacities (>250 mAh/g) are observed prior to cycle 5 that begin to fade upon extended cycling.

4 Assignment of peaks at ~121 ppm and ~43 ppm to PVDF in $^{1}H \rightarrow ^{13}C$ CPMAS NMR

As described in the main text, all $^{1}H \rightarrow ^{13}C$ CPMAS NMR experiments (both DNP and conventional SSNMR) show two resonances at ~121 ppm and ~43 ppm, not usually observed in the SEI, which we assign to the PVDF binder in the cathode composite. While the resonance at ~121 ppm may correspond to sp^2 hybridized carbons or fluorinated hydrocarbons (Leifer et al., 2011; Jin et al., 2018), the formation of sp^2 hybridized carbons has only been reported for cells cycled with FEC (Jin et al., 2018). The corresponding spectroscopic data for Li_2RuO_3 cells cycled in LP30 + 10% FEC are shown in Figures S11-S13 and are similar in this region to those measured in LP30 alone. Fluorinated hydrocarbons were observed in the SEI on graphite from the decomposition of PF_6^- and EC (Leifer et al., 2011), but these anodes also used PVDF as the binder material. PVDF contains a $-CH_2CF_2-$ repeat unit that can explain the presence of both the peak at 121 ppm and the peak 43 ppm (Montina et al., 2012).

In order to test our hypothesis that the ^{13}C signals at 121 ppm and 43 ppm come from fluorinated hydrocarbons in the PVDF binder, additional 1D $^{19}F \rightarrow ^{13}C$ CPMAS and ^{19}F SSNMR experiments were performed (Figures S5-S7). $^{19}F \rightarrow ^{13}C$ CPMAS NMR shows two ^{13}C resonances at ~44 ppm and ~122 ppm (Figure S6), consistent with the resonances assigned to PVDF in the DNP NMR and SSNMR data (Figures 2-4). Additionally, ^{19}F SSNMR further confirm this assignment, with ^{19}F resonances at −93 ppm and −114 ppm, which are indicative of the $RCH_2CF_xH_y$ environments present in PVDF (Figure S7) (Montina et al., 2012). Taken together, the $^{1}H \rightarrow ^{13}C$ CPMAS, $^{19}F \rightarrow ^{13}C$ CPMAS, $^{1}H \rightarrow ^{13}C$ HETCOR, and ^{19}F spin echo NMR provide compelling evidence that these resonances can be assigned to PVDF binder.

5 Assignment of fluorinated species in the CEI on Li_2RuO_3

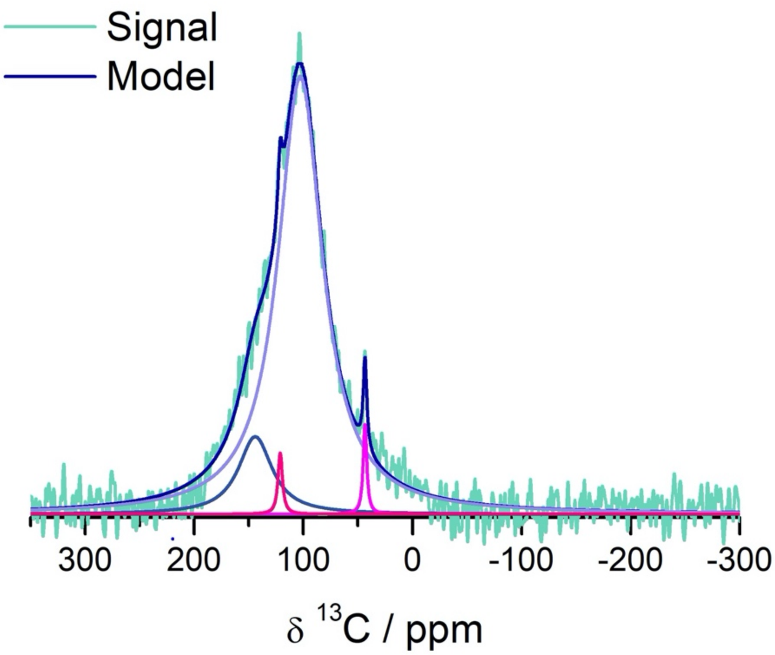

Figure S5: $^{19}F \rightarrow ^{13}C$ CPMAS SSNMR spectrum of a Li_2RuO_3 cathode cycled in LP30 + 10% FEC against Li metal for 27 cycles, disassembled in the discharged state at 2.0 V and recorded at 14.1 T at room temperature (conventional SSNMR). ^{19}F content in the probe caused a broad background signal in all ^{19}F and $^{19}F \rightarrow ^{13}C$ CPMAS data. Two peaks were used to fit the broad chemical shift anisotropy from the probe background (blue and light purple lines) and resonances from the sample are highlighted in the pink and red fitted peaks.

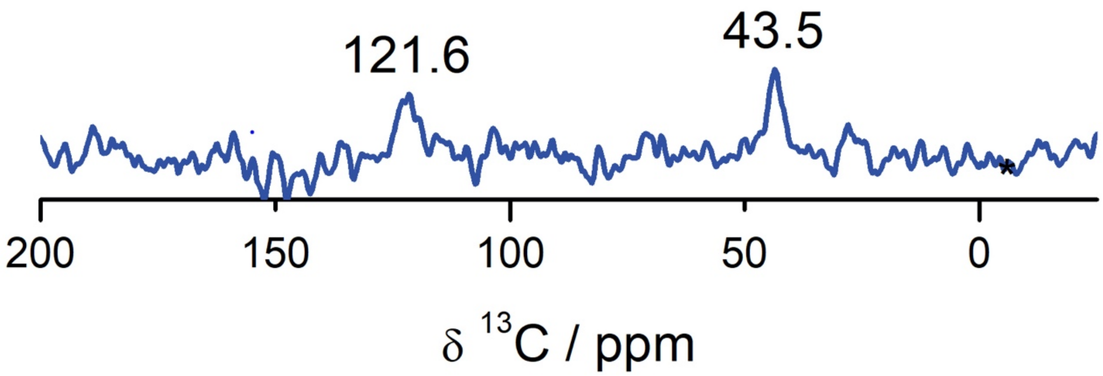

Figure S6: $^{19}F \rightarrow ^{13}C$ SSNMR spectrum in Figure S5 after baseline subtraction of the broad background peak.

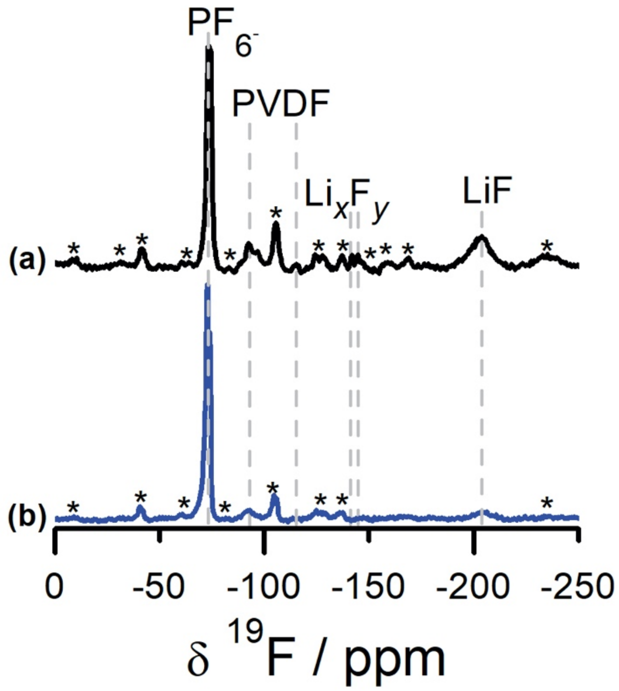

Figure S7: ^{19}F SSNMR spectra of Li_2RuO_3 cathodes cycled in LP30 against Li metal and disassembled (a, black) on charge after 100 cycles and (b, blue) on discharge after 27 cycles recorded at 14.1 T at room temperature (conventional SSNMR). Asterisks denote spinning sidebands. Peaks at −73 ppm and −203 ppm are assigned to PF_6^- and LiF, respectively. Peaks at −140 ppm and −144 ppm are both assigned to a lithiated fluoride phase Li_xF_y (Lebens-Higgins et al., 2019). Spectra are normalized to the PF_6^- peak in each individual spectrum (intensity scale is 0 to 1). Note that the intensity of LiF may not indicate a gradual buildup of LiF upon cycling because the relative amount of residual PF_6^- may change from sample to sample.

6 Electrochemical Impedance Spectroscopy (EIS)

6.1 Experimental Details

Potentiostatic EIS (PEIS) measurements were performed on a Biologic SP-150 potentiostat using a frequency range of 1 MHz to 0.1 Hz with a voltage perturbation of 10 mV. PEIS was recorded at the end of charge and the end of discharge at OCV after a rest period of 5 min for 9 cycles. Fitting was performed using the Biologic EC-Lab Zfit function.

6.2 Equivalent Circuit

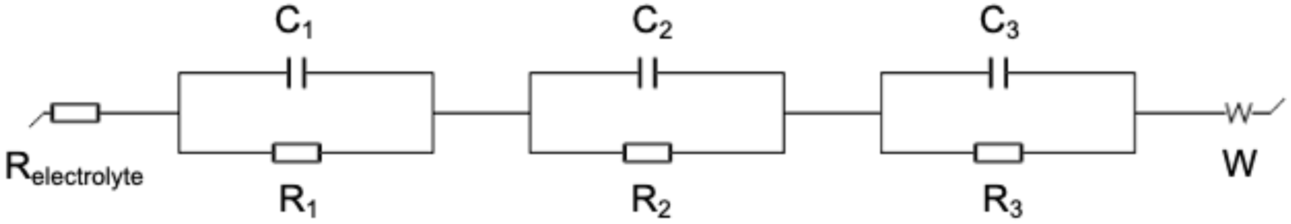

Figure S8: Equivalent circuit used for fitting where $R_{electrolyte}$ is resistance of the bulk electrolyte, R_1+R_2 represent contributions from the interfacial resistance, R_{CEI}, and R_3 is the charge transfer resistance, all paired with their associated capacitances (Thomas, 1985; Nobili et al., 2001; Zhang et al., 2006; Li et al., 2015; Liu et al., 2017). The Warburg element represents Li ion diffusivity (Thomas, 1985; Nobili et al., 2001; Zhang et al., 2006; Liu et al., 2017).

6.3 Fitting Results

Table S3: Equivalent circuit fit of EIS results (Figure S9) at various states of charge for Li_2RuO_3 cycled against Li in LP30.

Fit parameter	1st Charge	1st Discharge	2nd Charge	2nd Discharge	9th Charge	9th Discharge
$R_{electrolyte}$ (Ω)	20.3	16.3	11.7	12.3	11	11.1
R_1 (Ω)	1.39	1.927	1.2	1.117	1.502	0.9654
R_2 (Ω)	3.54	27.42	3.143	58.51	0.9642	49.39
R_{CEI} (Ω) (= R_1 + R_2)	4.93	29.35	4.343	59.63	2.466	50.36
R_3 (Ω)	331	6.826	2173	2.148	1311	0.870
C_1 (F)	4.347e-6	3.089e-6	6.515e-6	4.958e-6	0.1159e-3	3.089e-6
C_2 (F)	43.94e-6	0.1176	55.81e-6	0.03578	7.963e-6	0.100 8
C_3 (F)	7.937e-3	23.28e-6	6.051e-3	66.5e-6	6.878e-3	0.2633e-3
W (Ω $s^{-1/2}$)	14.75	25.92	6.442	17.3	9.447	17.2

EIS fitting results show specific trends in R_{CEI} and R_3 that vary based on the state of charge (i.e. whether the cell is in the discharged or charged state). The interfacial resistance (R_{CEI}) increases when measured in the discharged state (2.0 V) compared to the charged state (4.6 V), which is correlated to the increased deposition of organic electrolyte decomposition products (see main text for discussion). Conversely, the charge transfer resistance, R_3, greatly increases in the charged state compared to the discharged state, which is attributed poor kinetics of anionic redox due to the structural rearrangement necessary for oxygen loss from the lattice (Zhang et al., 2002; Assat et al., 2016; Liu et al., 2017; Nagao et al., 2019). On charge, R_3 becomes difficult to distinguish from the Warburg component.

Figure S9: EIS measurements (black) and corresponding fits (pink) of Li_2RuO_3 cycled against Li in LP30. EIS was conducted on the same sample but for the charged/discharged state for 9 cycles. These plots are magnified to show the $R_{CEI} = R_1 + R_2$ regions and omitting some of the Warburg diffusion.

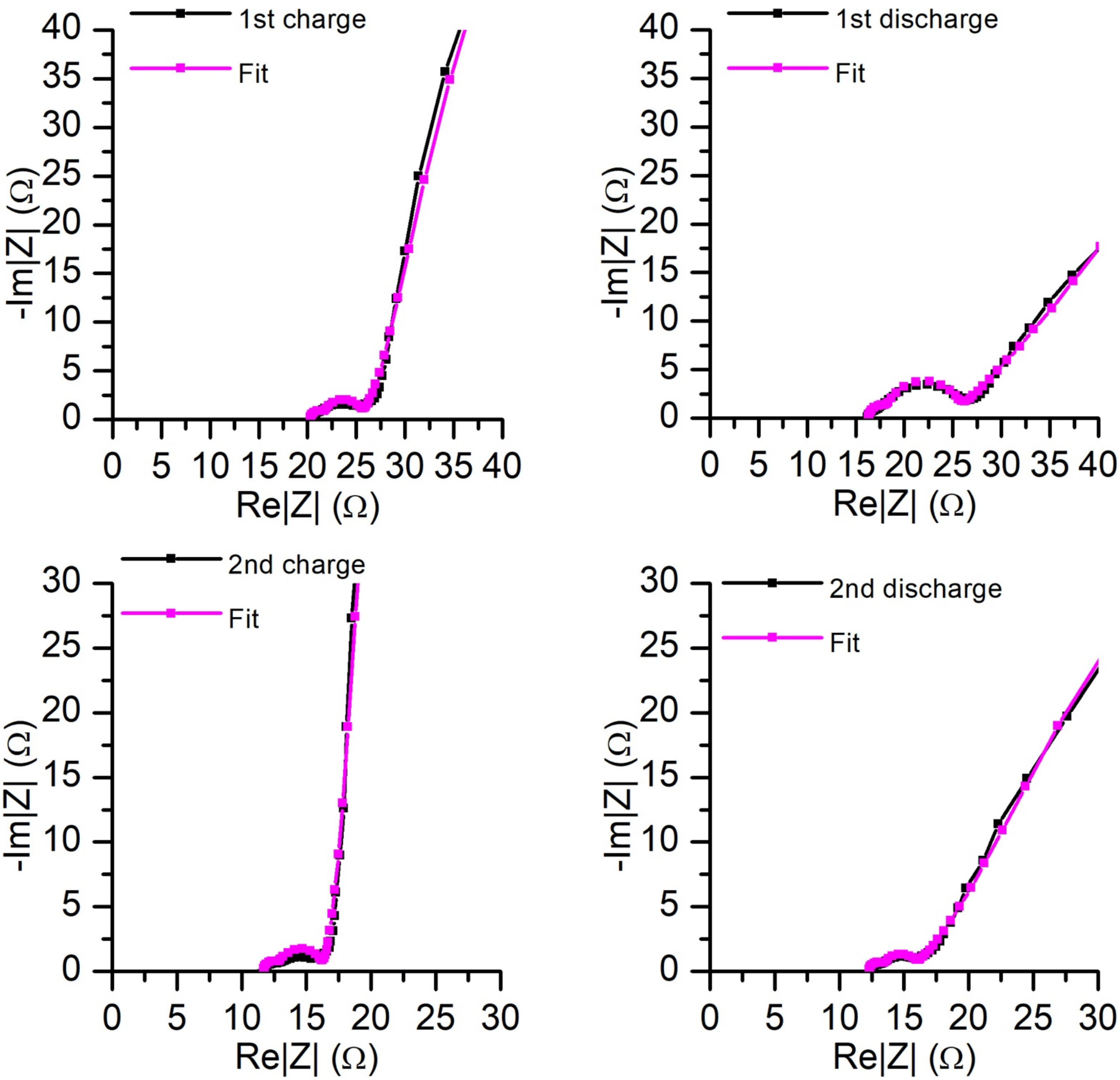

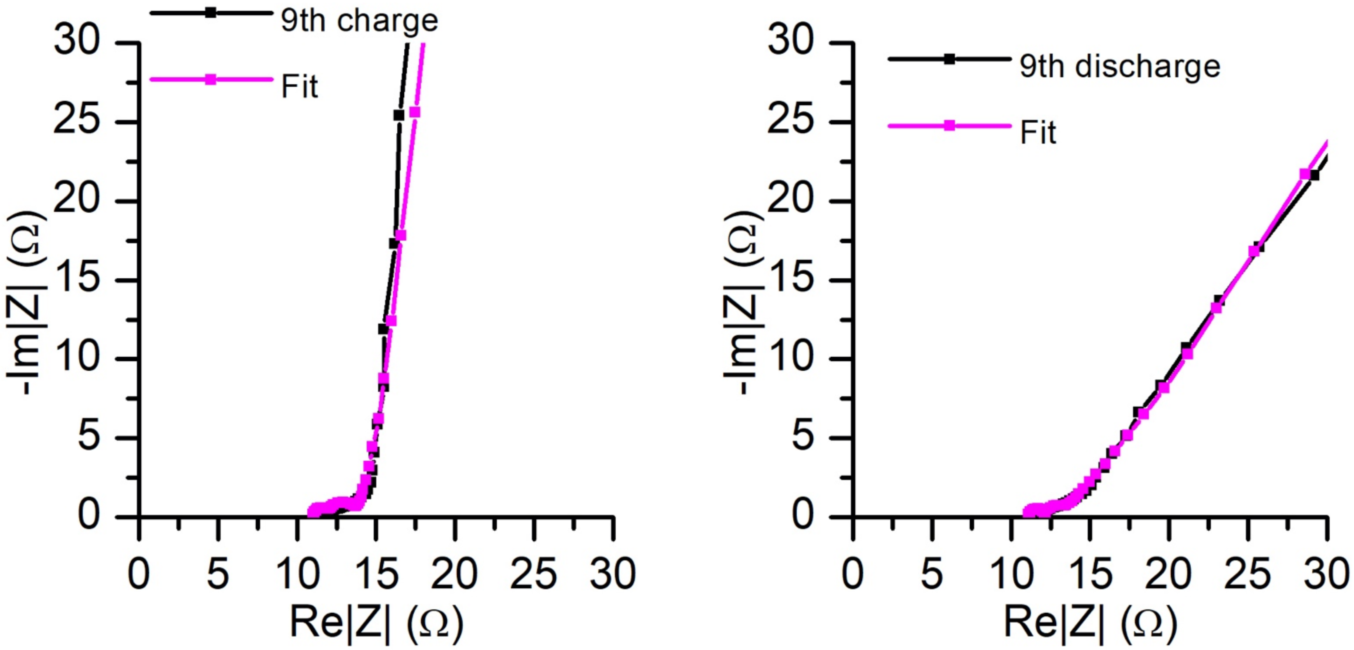
9th charge
Fit
-Im|Z| (Ω)
Re|Z| (Ω)
0
5
10
15
20
25
30
9th discharge
Fit
-Im|Z| (Ω)
Re|Z| (Ω)

7 Influence of fluoroethylene carbonate (FEC) on CEI composition

FEC has been extensively studied as both a cosolvent and an additive in Li-ion battery electrolytes (Markevich et al., 2017). Previous work has shown that FEC notably improves capacity retention and reduces voltage fade by altering the electrode-electrolyte interphase of graphitic anodes (Mogi et al., 2002; Profatilova et al., 2009), Si anodes (Etacheri et al., 2011; Nakai et al., 2011; Xu et al., 2015; Jin et al., 2017), and cathode materials (Markevich et al., 2014; Li et al., 2015). However, the molecular mechanisms underpinning these performance enhancements is debated in the literature and may vary for different systems. Analyses of the CEI on Li_2RuO_3 cathodes cycled in FEC-containing electrolyte solutions indicate that FEC significantly decreases the amount of organic species in the CEI, such as alkyl carbonates and PEO-type polymers (Figures S10 and S11). These findings support that FEC may enhance CEI stability by mitigating continuous electrolyte decomposition at early stages of electrochemical cycling (Figure S13). However, Li_2RuO_3|Li cells containing FEC still exhibit severe capacity fade by cycle 100, indicating that the alternative organic CEI that forms with FEC present is also not stable and/or electrode degradation contributes to capacity loss.

7.1 NMR Analysis

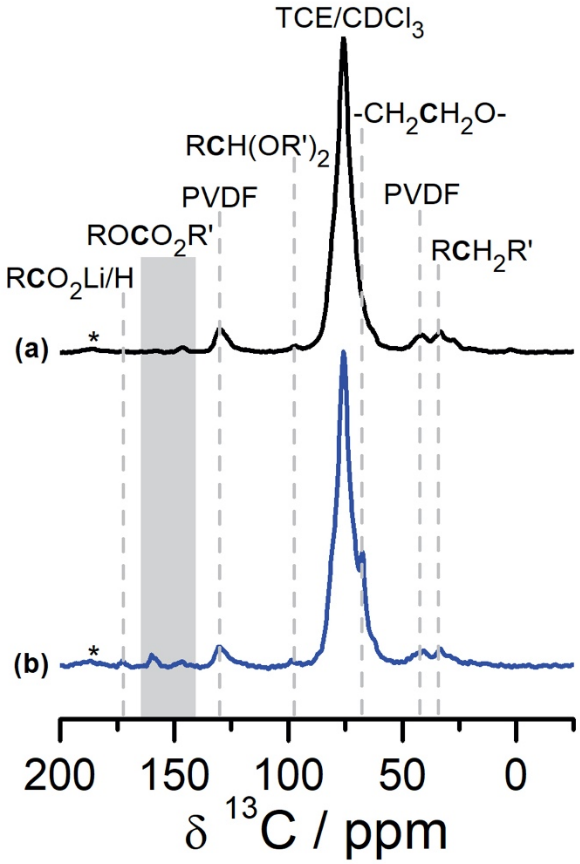

Figure S10: $^{1}H \rightarrow ^{13}C$ CPMAS DNP NMR spectra of Li_2RuO_3 cathodes cycled in (a) LP30 + 10% FEC and (b) LP30 against Li metal, disassembled in discharged state of 2.0 V after 27 cycles and recorded at 9.4 T and T = 92 K. The grey rectangle is to denote the alkyl carbonate ($ROCO_2R'$) region that corresponds to peaks at 148 and 160 ppm. Asterisks denote spinning side bands. The LP30 cell corresponds to Sample 1 and the LP30 + 10% FEC cell corresponds to Sample 2 in Table S1.

Figure S10 compares the CEI composition of a cell cycled in LP30 to a cell cycled in LP30 + FEC. As expected, both cells show ^{13}C resonances that correspond to PVDF binder in the cathode composite. The FEC-containing electrolyte does not show a peak at ~68 ppm for PEO-type species in the CEI. Additional SSNMR experiments confirm that the FEC sample has no PEO peak on discharge (Figure S11b, black), whereas this resonance is prominent in the LP30 sample (Figure S11b, blue) at 2.0 V. After 100 cycles, a small PEO peak emerges at the end of charge for FEC-containing electrolytes (Figure S11a, black), indicating that FEC mitigates the formation of PEO-type polymers during electrochemical cycling. Comparison of the carbonate regions between samples with and without FEC indicates that in the absence of FEC, carbonate electrolytes consistently decompose into alkyl carbonates ($ROCO_2R'$) on the cathode surface (Figures S10b and S11b, blue) whereas the formation of these species is largely hindered when FEC is present (Figures S10a and S11b, black). In both FEC and non-FEC cases, these alkyl carbonates are stripped from the cathode surface during charging (Figure S11a). A small peak around ~25 ppm in the DNP (Figure S10a) appears in the FEC-containing

sample. This peak may correspond to LBDC or Li succinate (RCH_2CH_2R') (Leskes et al., 2017) that can be assigned using 2D NMR correlation; however, this peak had too low of SNR to appear in the HETCOR spectrum. The amount of Li acetate present in different samples (^{13}C resonances at 177 ppm and 18 ppm, Figure S11b, black and Figure S10a) is difficult to reproduce, indicating that the formation of this species may vary cell-to-cell.

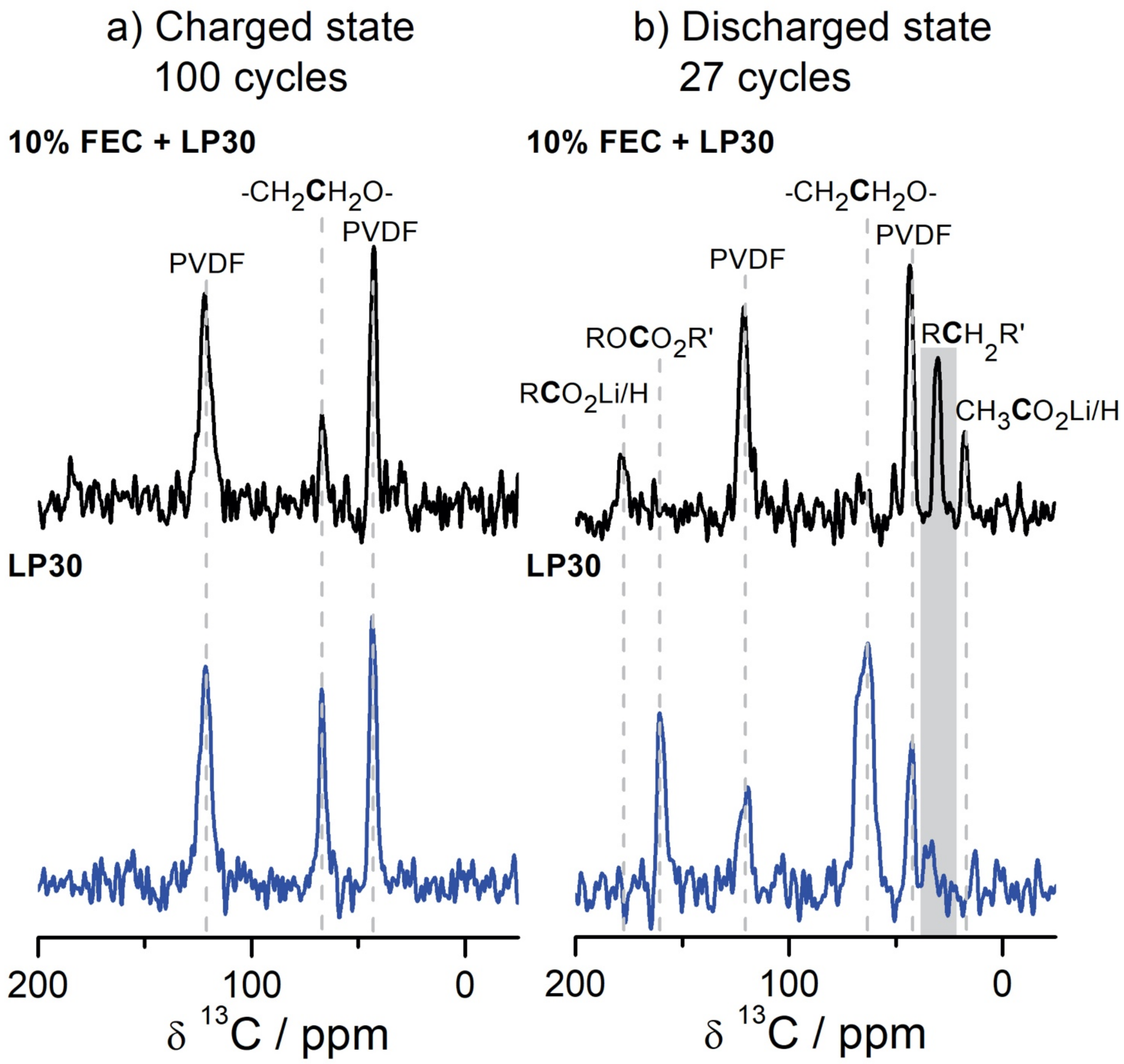

Figure S11: $^1H \rightarrow ^{13}C$ CPMAS SSNMR spectra of Li_2RuO_3 cathodes cycled in LP30 (blue, bottom) or LP30 + 10% FEC (black, top) against Li metal and disassembled in the (a) charged state of 4.6 V and (b) discharged state of 2.0 V and recorded at 14.1 T at room temperature (conventional SSNMR). The grey rectangle is used for labeling of the RCH_2R' region at 31-36 ppm which may be assigned to LBDC or Li succinate (Leskes et al., 2017).

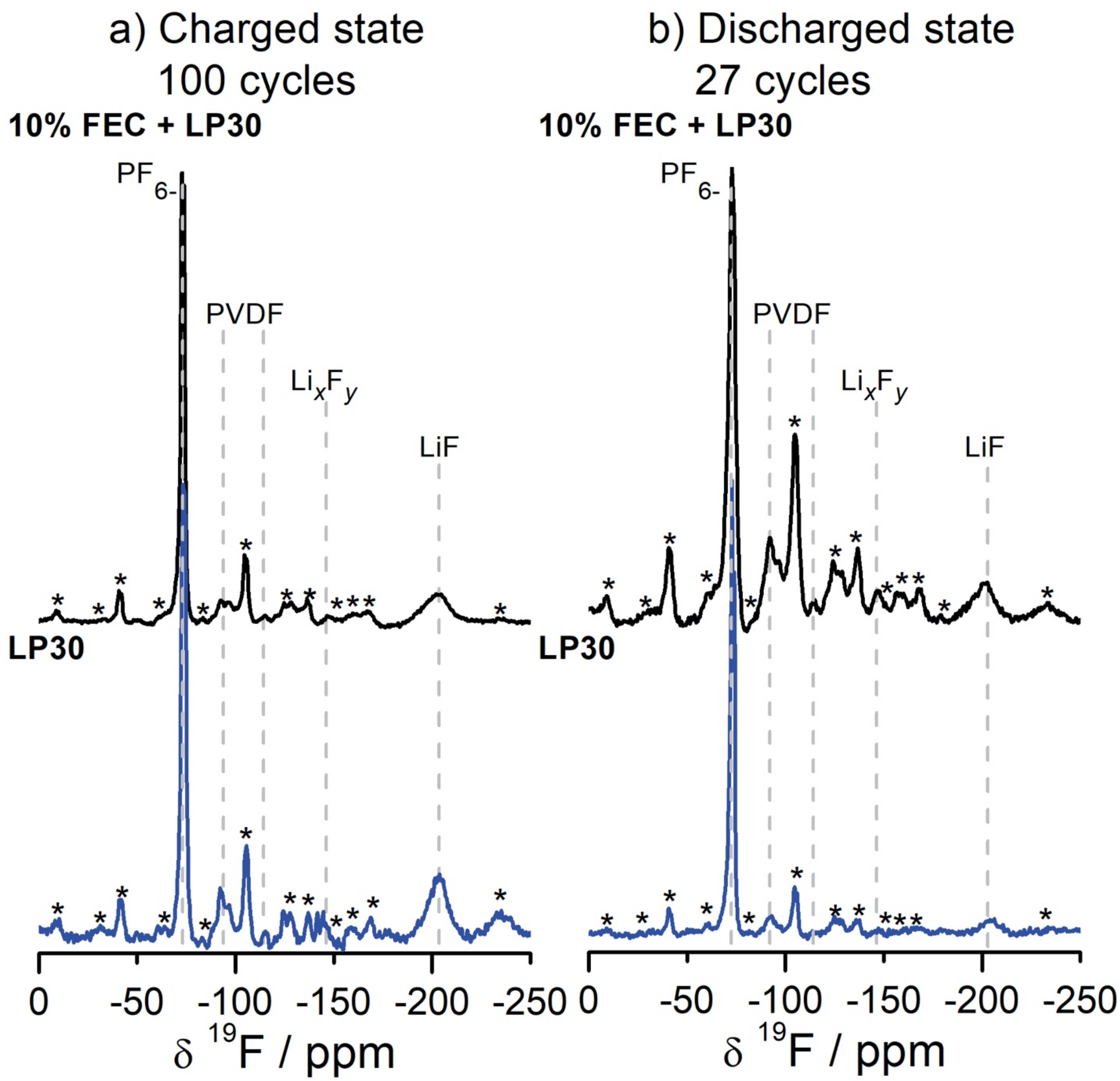

Figure S12: ^{19}F SSNMR spectra of the CEI on Li_2RuO_3 cycled against Li metal in LP30 (blue, bottom) or LP30 + 10% FEC (black, top). The cells shown in (a) were disassembled in the charged state at 4.6 V after 100 cycles. The cells shown in (b) were disassembled in the discharged state at 2.0 V after 27 cycles. Spectra are normalized to the PF_6^- peak in each individual spectrum (intensity scale is 0 to 1). Asterisks denote spinning side bands. Similar fluorinated compounds are found in the CEI of cells cycled with and without FEC content.

7.2 Specific charge capacity vs cycle number for cells with and without FEC

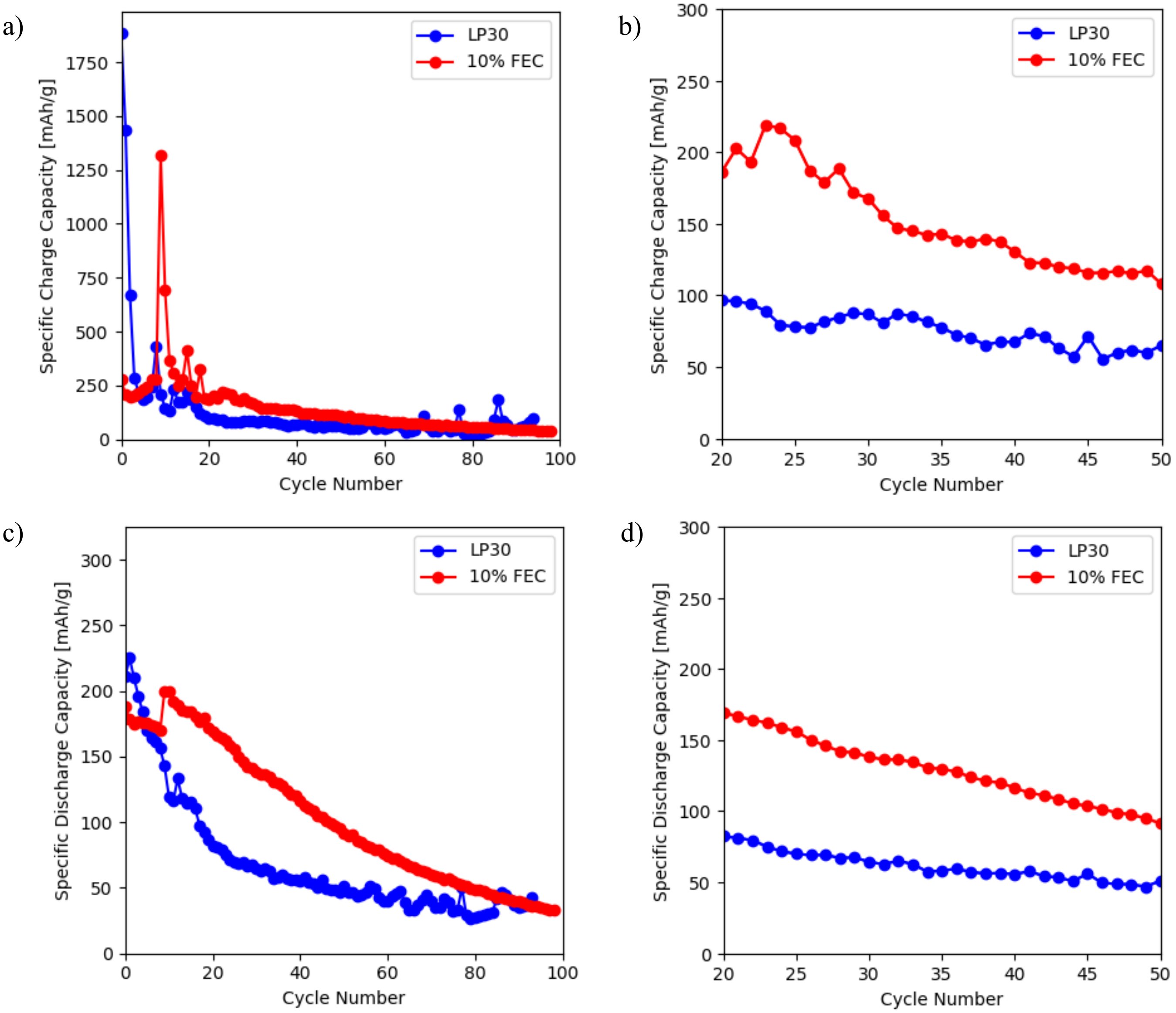

Figure S13: Specific (a, b) charge and (c, d) discharge capacities as a function of cycle number for Li_2RuO_3 cathodes cycled against Li metal in LP30 (blue) and LP30 + 10% FEC (red) at C/30. The LP30 cell corresponds to the cell analyzed in Figure 4 (a, black) and the 10% FEC cell corresponds to the cell analyzed in Figure S10 (a, black) and S11 (a, black). Note that the charge capacity spikes in (a) are from continuous cell charging at high voltages just before reaching 4.6 V, possibly due to side reactions with the electrolyte. The FEC cell capacity is slightly improved compared to LP30 alone, but both electrolytes show similar capacity fading, indicating that altering the composition of the organic CEI does not dramatically improve charge capacity of Li_2RuO_3|Li cells.

References

Assat, G., Delacourt, C., Corte, D. A. D., and Tarascon, J.-M. (2016). Editors' Choice—Practical Assessment of Anionic Redox in Li-Rich Layered Oxide Cathodes: A Mixed Blessing for High Energy Li-Ion Batteries. *J. Electrochem. Soc.* 163, A2965–A2976. doi:10.1149/2.0531614jes.

Etacheri, V., Haik, O., Goffer, Y., A. Roberts, G., C. Stefan, I., Fasching, R., et al. (2011). Effect of Fluoroethylene Carbonate (FEC) on the Performance and Surface Chemistry of Si-Nanowire Li-Ion Battery Anodes. *Langmuir* 28, 965–976. doi:10.1021/la203712s.

Jin, Y., H. Kneusels, N.-J., C. M. M. Magusin, P., Kim, G., Castillo-Martínez, E., E. Marbella, L., et al. (2017). Identifying the Structural Basis for the Increased Stability of the Solid Electrolyte Interphase Formed on Silicon with the Additive Fluoroethylene Carbonate. *J. Am. Chem. Soc.* 139, 14992–15004. doi:10.1021/jacs.7b06834.

Jin, Y., H. Kneusels, N.-J., E. Marbella, L., Castillo-Martínez, E., C. M. M. Magusin, P., S. Weatherup, R., et al. (2018). Understanding Fluoroethylene Carbonate and Vinylene Carbonate Based Electrolytes for Si Anodes in Lithium Ion Batteries with NMR Spectroscopy. *J. Am. Chem. Soc.* 140, 9854–9867. doi:10.1021/jacs.8b03408.

Lebens-Higgins, Z. W., Halat, D. M., Faenza, N. v., Wahila, M. J., Mascheck, M., Wiell, T., et al. (2019). Surface Chemistry Dependence on Aluminum Doping in Ni-rich $LiNi_{0.8}Co_{0.2-y}Al_yO_2$ Cathodes. *Sci. Rep.* 9, 1–12. doi:10.1038/s41598-019-53932-6.

Leifer, N., Smart, M. C., Prakash, G. K. S., Gonzalez, L., Sanchez, L., Smith, K. A., et al. (2011). ^{13}C Solid State NMR Suggests Unusual Breakdown Products in SEI Formation on Lithium Ion Electrodes. *J. Electrochem. Soc.* 158, A471. doi:10.1149/1.3559551.

Leskes, M., Kim, G., Liu, T., L. Michan, A., Aussenac, F., Dorffer, P., et al. (2017). Surface-Sensitive NMR Detection of the Solid Electrolyte Interphase Layer on Reduced Graphene Oxide. *J. Phys. Chem. Lett.* 8, 1078–1085. doi:10.1021/acs.jpclett.6b02590.

Li, Y., Lian, F., Ma, L., Liu, C., Yang, L., Sun, X., et al. (2015). Fluoroethylene Carbonate as Electrolyte Additive for Improving the electrochemical performances of High-Capacity $Li_{1.16}[Mn_{0.75}Ni_{0.25}]_{0.84}O_2$ Material. *Electrochim. Acta* 168, 261–270. doi:10.1016/j.electacta.2015.04.030.

Liu, S., Wang, J., Tian, Z., Li, Q., Tian, X., Cui, Y., et al. (2017). Chromium doped Li_2RuO_3 as a positive electrode with superior electrochemical performance for lithium ion batteries. *Chem. Commun.* 53, 11913–11916. doi:10.1039/c7cc07545f.

Markevich, E., Salitra, G., and Aurbach, D. (2017). Fluoroethylene Carbonate as an Important Component for the Formation of an Effective Solid Electrolyte Interphase on Anodes and Cathodes for Advanced Li-Ion Batteries. *ACS Energy Lett.* 2, 1337–1345. doi:10.1021/acsenergylett.7b00163.

Markevich, E., Salitra, G., Fridman, K., Sharabi, R., Gershinsky, G., Garsuch, A., et al. (2014). Fluoroethylene Carbonate as an Important Component in Electrolyte Solutions for High-Voltage

Lithium Batteries: Role of Surface Chemistry on the Cathode. *Langmuir* 30, 7414–7424. doi:10.1021/la501368y.

Mogi, R., Inaba, M., Jeong, S.-K., Iriyama, Y., Abe, T., and Ogumi, Z. (2002). Effects of Some Organic Additives on Lithium Deposition in Propylene Carbonate. *J. Electrochem. Soc.* 149, A1578. doi:10.1149/1.1516770.

Montina, T., Wormald, P., and Hazendonk, P. (2012). ^{13}C Solid-State NMR of the Mobile Phase of Poly(vinylidene fluoride). *Macromolecules* 45, 6002–6007. doi:10.1021/ma3013477.

Nagao, K., Sakuda, A., Nakamura, W., Hayashi, A., and Tatsumisago, M. (2019). Fast Cationic and Anionic Redox Reactions in Li_2RuO_3-Li_2SO_4 Positive Electrode Materials. *ACS Appl. Energy Mater.* 2, 1594–1599. doi:10.1021/acsaem.8b02163.

Nakai, H., Kubota, T., Kita, A., and Kawashima, A. (2011). Investigation of the Solid Electrolyte Interphase Formed by Fluoroethylene Carbonate on Si Electrodes. *J. Electrochem. Soc.* 158, A798. doi:10.1149/1.3589300.

Nobili, F., Tossici, R., Croce, F., Scrosati, B., and Marassi, R. (2001). Electrochemical ac impedance study of LixNi0.75Co0.25O2 intercalation electrode. *J. Power Sour.* 94, 238–241. doi:10.1016/S0378-7753(00)00592-9.

Profatilova, I. A., Kim, S. S., and Choi, N. S. (2009). Enhanced thermal properties of the solid electrolyte interphase formed on graphite in an electrolyte with fluoroethylene carbonate. *Electrochim. Acta* 54, 4445–4450. doi:10.1016/j.electacta.2009.03.032.

Thomas, M. G. S. R., Bruce, P.G., and Goodenough, J.B. (1985). AC Impedance Analysis of Polycrystalline Insertion Electrodes: Application to $Li_{1-x}CoO_2$. *J. Electrochem. Soc.* 132, 1521. doi:10.1149/1.2114158.

Xu, C., Lindgren, F., Philippe, B., Gorgoi, M., Björefors, F., Edström, K., et al. (2015). Improved Performance of the Silicon Anode for Li-Ion Batteries: Understanding the Surface Modification Mechanism of Fluoroethylene Carbonate as an Effective Electrolyte Additive. *Chem. Mater.* 27, 2591–2599. doi:10.1021/acs.chemmater.5b00339.

Zhang, S. S., Xu, K., and Jow, T. R. (2002). Formation of solid electrolyte interface in lithium nickel mixed oxide electrodes during the first cycling. *Electrochem. Solid-State Lett.* 5, A92. doi:10.1149/1.1464506.

Zhang, S. S., Xu, K., and Jow, T. R. (2006). EIS study on the formation of solid electrolyte interface in Li-ion battery. *Electrochim. Acta* 51, 1636–1640. doi:10.1016/j.electacta.2005.02.137.
